# Equivalency of risk for a modified health endpoint: a case from recreational water epidemiology studies

**DOI:** 10.1186/1471-2458-13-459

**Published:** 2013-05-10

**Authors:** Larry J Wymer, Timothy J Wade, Alfred P Dufour

**Affiliations:** 1 United States Environmental Protection Agency, National Exposure Research Laboratory, 26 W. Martin Luther King Drive, Cincinnati, OH 45268, USA; 2 United States Environmental Protection Agency, National Health Effects Research Laboratory, 109 T.W. Alexander Drive, Research Triangle Park, NC 27709, USA

**Keywords:** Recreational water quality, Swimming-related illness, Gastrointestinal illness, Epidemiological study

## Abstract

**Background:**

The United States Environmental Protection Agency (USEPA) and its predecessors have conducted three distinct series of epidemiological studies beginning in 1948 on the relationship between bathing water quality and swimmers’ illnesses. Keeping pace with advances in microbial technologies, these studies differed in their respective microbial indicators of water quality. Another difference, however, has been their specific health endpoints. The latest round of studies, the National Epidemiological Assessment of Recreational (NEEAR) Water studies initiated in 2002, used a case definition, termed “NEEAR GI illness” (NGI), for gastrointestinal illness corresponding closely to classifications employed by contemporary researchers, and to that proposed by the World Health Organization. NGI differed from the previous definition of “highly credible gastrointestinal illness” (HCGI) upon which the USEPA’s 1986 bathing water criteria had been based, primarily by excluding fever as a prerequisite.

**Methods:**

Incidence of NGI from the NEEAR studies was compared to that of HCGI from earlier studies. Markov chain Monte Carlo method was used to estimate the respective beta binomial probability densities for NGI and HCGI establish credible intervals for the risk ratio of NGI to HCGI.

**Results:**

The ratio of NGI risk to that of HCGI is estimated to be 4.5 with a credible interval 3.2 to 7.7.

**Conclusions:**

A risk level of 8 HCGI illnesses per 1000 swimmers, as in the 1986 freshwater criteria, would correspond to 36 NGI illnesses per 1000 swimmers. Given a microbial DNA-based (qPCR) water quality vs. risk relationship developed from the NEEAR studies, 36 NGI per 1000 corresponds to a geometric mean of 475 qPCR cell-equivalents per 100 ml.

## Background

The interest in setting water quality standards for bathing beach waters extends back to the early part of the 20th century. The microbiological quality of recreational waters was first discussed in the United States as early as 1922 by the American Public Health Association’s Committee on Bathing Beaches [1]. The Committee Report in 1924 [2] concluded that there was not enough evidence to develop bathing water standards for natural waters. In June, 1933 the Joint Committee on Bathing Places was formed and in their first report noted that because of the great lack of epidemiological information no bacterial standards were adopted [3]. The reluctance to propose bacterial standards for outdoor bathing places extended from 1936 to1957 [4-6]. Even as late as 1957 the Committee stated that very little reliable data were available to implicate bathing places in the spread of disease [6].

In 1948 the US Public Health Service (US PHS) began a series of epidemiological studies addressing the relationship between bathing water quality and illness in swimmers exposed to the beach waters [7]. These studies looked at multiple symptoms that might be associated with respiratory, eye, ear, skin and gastrointestinal (GI) infections. Fecal contamination in the waters was measured by total coliform bacteria. The data collected during the US PHS studies was used in 1968 by the National Technical Advisory Committee (NTAC) to develop recommendations for bathing beach criteria [8]. The first criterion for recreational waters proposed that the geometric mean of five samples taken over a 30 day period should not exceed 200 fecal coliforms per hundred milliliters. The case definition or health endpoint used during the US PHS studies was any one or more of the following: stomach or intestinal upset, diarrhea, vomiting or nausea [9] (see Table 1). It should be noted that fecal coliforms were not used to measure water quality during the US PHS studies, but the NTAC used bacterial indicator data from Ohio River samples collected in the 1960s, which showed that the ratio of fecal coliform to total coliforms was approximately 1:5. Thus, the mean density of total coliforms associated with a detectable GI illness rate in the US PHS studies (which was approximately 2300 per hundred milliliters) was converted to 400 fecal coliforms and then lowered to 200 fecal coliforms per hundred milliliters in the belief that this would result in a zero GI illness rate in swimmers.

**Table 1:** Gastrointestinal illness case definitions: US studies

		**Case definition^1^**			
**Definition**	**Description**	**Diarrhea**	**Vomiting**	**Stomachache**	**Nausea**
US PHS McCabe [9]	Gastrointestinal Illness	Any	Any	Any	Any
EPA-1986 Cabelli [10]	Highly Credible Gastrointestinal Illness	w/fever or disabling	Any	w/fever	w/fever
NEEAR Wade et al. [35]	Gastrointestinal Illness	3 episodes in 24 h.	Any	Disabling or with nausea	Disabling or with stomachache

^1^Presence of any single symptom. Any indicated, concomitant condition (e.g. “w/fever”) is required to be present in addition to the primary symptom.

Less than four years later the newly formed US Environmental Protection Agency (US EPA) began another series of epidemiological studies that were designed to improve on some of the shortcomings of the US PHS studies. A major improvement involved the use of a new case definition which included combinations of symptoms (see Table 1), thus providing a more reliable health endpoint termed “highly credible gastrointestinal illness” (HCGI). These studies also included new methods for measuring water quality. Membrane filter methods for Escherichia coli and enterococci were introduced as new measures of water quality for bathing beach waters [9-11]. Data from the US EPA studies were used in 1986 to develop new water quality criteria for marine and freshwater beaches. The epidemiological studies showed that in marine waters the density of enterococci had the strongest relationship to GI illness in swimmers, while in fresh waters both E. coli and enterococci densities showed a strong relationship to swimming-associated GI illness. Rather than developing criteria based on a new acceptable risk level, the 1968 criteria were translated to new criteria by setting a simple algebraic proportion equation, where the proportion of the unknown criteria to the average density of the new indicator is equal to the proportion of the fecal coliform criteria (200 fecal coliform per 100 ml) to the average density of the fecal coliforms [12]. The resulting criteria values were related to health risk values using the regression equations for health on water quality for both marine and fresh recreational waters.

In 2002 another series of epidemiological studies was initiated by the US EPA titled, the National Epidemiological and Environmental Assessment of Recreational (NEEAR) water studies. The objective of the new studies was to determine the relationship between swimming-associated GI illness and water quality measured with new methods which produce same-day results. The studies differed from the early EPA epidemiology studies in three significant ways:

1. A more contemporary case definition for gastroenteritis was used. The new health endpoint for GI illness did not include fever as a necessary requirement (Table 1). One reason for the change in illness definition was to allow consideration of viral illness (such as norovirus) which may not produce high fever in combination with GI symptoms. This case definition is more similar to that suggested by a group of international experts who proposed that a uniform international definition should include vomiting or diarrhea constituting greater than or equal to three soft stools in a 24 hour period without a requirement of fever [13], as well as by the World Health Organization [14]. The revised definition is also similar to definitions used in other epidemiology studies of GI illness in relation to waterborne exposures in North America [15-17]. This case definition is labeled NEEAR gastrointestinal illness (NGI) to distinguish it from the case definition used in the earlier EPA studies. In addition, the first series of studies had different follow up period over which illness was assessed (8–10 days for the 1986 studies, and 10–12 days for the NEEAR studies).

2. The data were analyzed using a different approach than that used in 1986. Water quality measures were grouped by day, rather than by summer trial as in the early EPA studies. Furthermore, the current analysis used regression modeling techniques which allowed consideration and control for individual characteristics such as age, sex and other demographic characteristics.

3. Water quality was measured using a quantitative polymerase chain reaction method [18]. The analyte for this method is the DNA obtained from enterococci in a 100 mL water sample. Target DNA from live and dead enterococci cells is measured by qPCR, unlike culture methods, which only measure viable and culturable cells. The molecular indicator is less influenced by environmental stresses and its degradation characteristics in water are more similar to that of viral and protozoan pathogens, both of which have been shown to persist in water environments longer than the currently used fecal indicator bacteria [19,20].

In 1986, criteria were developed based on data from beach waters that met a fecal coliform standard [21]. Coliforms, and thus fecal coliforms, have been shown to have widely different die-off rates in marine and freshwater. The T_90_ (time for 90% of the coliforms to die) for fresh water was about 26 hours, whereas the T_90_ in marine waters was about 2.5 hours [12]. This difference was thought to influence the fecal coliform-illness relationship in marine waters where higher swimming-associated illness rates were observed than those among freshwater swimmers at comparable coliform levels. Similar swimming-associated GI illness rate differences were observed between marine and freshwater swimmers in Europe and the UK [22], where fecal coliforms also were used to measure water quality. Since 1986 the US EPA has recommended the use of enterococci as a measure of water quality for both marine and fresh bathing beach waters. Enterococci show much slower die-off rates in marine waters than coliform indicator bacteria [23,24].

By removing the requirement of fever and by including a longer follow up period, the NGI definition is broader compared to HCGI and as a result, the background rate of NGI is higher than HCGI. The effect of relaxing the stringency and broadening the case definition has been described by Wiedenmann [25]. He examined the same non-swimming population using three different case definitions (Table 2). He showed that by eliminating the requirement for consideration of stool frequency from the case definition the gastrointestinal illness rate increases by a factor of two, from 14 per thousand to 28 per thousand. Similarly, removing the fever symptom from the case definition increased the illness rate by a factor of 3.7, from 14 per thousand to 52 per thousand. Multiple case definitions also were studied simultaneously in a non-swimming population by van Asperen (Table 2) and her co-investigators [26]. They examined the rates of GI illness using case definitions from the US EPA studies [9], the United Kingdom studies [27], and the Netherlands studies [26]. The EPA case definition was the most stringent, followed by the UK and the Netherlands. The GI illness rate increased from eight per thousand to 17 per thousand to 21 per thousand as the stringency of the case definition was relaxed. These studies would predict that there would be a similar increase in the GI illness rate between the early EPA studies and the NEEAR study due to a change in the case definition.

**Table 2:** Effect of case definition on gastrointestinal illness rate

**Reference**	**Study**	**Case definition^1^**				**Non-swimmer illness rate per 1000**
		**Diarrhea**	**Vomiting**	**Stomachache**	**Nausea**	
Wiedenmann [25]	UK^2^	3 episodes in 24 hours	Any	w/fever	w/fever	14
	UKwf^3^	Any	Any	w/fever	w/fever	28
	NL2^4^	Any	Any	Any	Any	52
van Asperen [26]	USEPA^5^	w/fever or disabling	Any	w/fever	w/fever	8
	UK^2^	3 episodes in 24 hours	Any	w/fever	w/fever	17
	NL2^4^	Any	Any	Any	Any	21

^1^Presence of any single symptom. Any indicated concomitant condition (e.g. “w/fever”) is s required to be present in addition to the primary symptom. ^2^ Kay, [27]. ^3^ Wiedenmann, [25]. ^4^ van Asperen, [26]. ^5^ Cabelli, [10].

The Beaches Environmental Assessment and Coastal Health (BEACH) Act of 2000 [28] mandated that studies be conducted and new recreational water criteria developed based on these studies that are “as protective as” that which existed in the 1986 criteria. Thus, swimming-related risk of illness relative to the baseline level for new criteria should be the same as, or better than, it was when the 1986 criteria were established, keeping mindful of the fact that the mean baseline illness rate may change over time even for a fixed case definition of illness. It is, then, our objective to develop an approach to translate the recreational water tolerable illness rate for pre-1986 HCGI to an equivalent illness rate for the contemporary NGI definition that takes into account all factors that may affect the difference, including temporal effects, and use this equivalence to convert the new equivalent illness rate to an Enterococcus qPCR guideline value based on the NEEAR data. In addition, we present a regression model utilizing all NEEAR study data to estimate NGI illness risk and to provide a basis for interpreting the results of the translation. Much of the information in this paper has appeared online as an appendix to the USEPA 2012 recreational water criteria [29]. Here we present technical details, including an estimation of the respective distributions of historical HCGI and contemporary NGI background illness rates and related statistical analyses along with expanded discussion of these results.

## Methods

We use the baseline GI illness rates (rates among non-swimmers) in the USEPA and NEEAR studies to investigate the relationship between the HCGI and NGI case definitions and any potential changes in the disease burden. Illnesses among swimmers are not considered because the relationship between HCGI and NGI symptom rates among swimmers may be influenced by systematic disparity in their exposure to contaminated bathing water. Differences in reported HCGI and NGI illnesses among non-swimmers in these respective studies will be influenced by differences not only in case definition, but also in the length of follow up period, temporal effects, and even changes in health awareness over the approximately 30 years that intervene these two sets of studies. The latter effects are confounded with case definition, but it is the overall combined effect that is relevant in determining an illness rate for the current NGI definition that is equivalent to that of the former HCGI definition. The non-swimmer illness rates of the two populations will give the best estimate of any inherent changes in the background illness rates that may have occurred between studies conducted in the mid to late 1970’s which form the basis for the 1986 Criteria and the studies conducted during the years 2002–2009, and provide an unbiased estimate of the effect of changing the case definition for gastroenteritis.

Non-swimmer illness rates are seen to vary beach-by-beach both in the old studies using HCGI and the NEEAR study using NGI as the case definition. These geographic differences between the respective studies are accounted for in the form of random spatial/temporal effects in our model of HCGI case definition on NGI case definition. As commonly done for data consisting of binomial realizations from several independent subpopulations, where each subpopulation may be associated with a different probability of “success,” a beta-binomial model is used (see, for example, [30]). Background illness rates among the various beaches are assumed to follow a beta distribution, which has the desirable properties of being bounded between zero and one, and can assume a range of shapes capable of describing a variety of distributions of illness rates. Within a beach the number of illnesses observed among non-swimmers is assumed to be described by the binomial with probability of illness unique to that beach. Parameters for the beta distribution are estimated separately for HCGI and NGI using the VGAM package (version.8-1; T.W.Yee, University of Auckland, New Zealand), an add-on for the R computer software for Windows (version 2.8.1; R Development Core Team, 2009). Because of the relatively small number of individual beaches used in these studies, Bayesian confidence intervals (“credible intervals”) for the mean probabilities of HCGI and NGI [=α/(α + β) from the beta-binomial parameters] and risk ratio of NGI to HCGI were evaluated via Markov Chain Monte Carlo (MCMC) estimation using WinBUGS version 1.4.3 [31]. MCMC uses simulation techniques to sample the distribution of possible values for these probabilities based on the observed data [32].

Once the equivalence between NGI and HCGI has been established based on non-swimmer data from the NEEAR and 1986 studies, respectively, we can translate the tolerable HCGI risk of the 1986 criteria to an equivalent NGI health risk. Tolerable risk was defined in the 1986 criteria in terms of the swimming-associated illness (SAI) rate per 1000 swimmers (8 HCGI illnesses for freshwater and 19 for marine waters) [33].

Key to translating the tolerable illness level from the 1986 criteria to an equivalent illness risk level for data from the NEEAR study is the relative risk (RR) expression. Relative risk is defined as the ratio of the illness rate in exposed individuals to the illness rate in non-exposed individuals. The RR, then, is related to the increase in risk in an exposed population. In our case, the relationship is given by RR = (NS + SAI)/NS, where NS is the non-exposed illness rate (represented by non-swimmers). That is, the tolerable RR is given by the total tolerated swimmers’ risk, which is the sum of the non-exposed illness risk (NS) plus SAI, divided by the non-exposed risk, NS. The approach is to make the relative risk of NGI as defined in the NEEAR studies equivalent to the relative risk of HCGI in the 1986 EPA studies.

The translation algorithm for converting the 1986 criteria illness rate to an equivalent illness rate from the NEEAR data may be described as a two step process.

Step 1. Calculate the relative risk (RR) for the 1986 criterion value:

$${\mathrm{RR}}_{\mathrm{HCGI}}=\left({\mathrm{NS}}_{\mathrm{HCGI}}{+\mathrm{SAI}}_{\mathrm{HCGI}}\right)/{\mathrm{NS}}_{\mathrm{HCGI}}$$

Step 2. Set SAI_NGI_ to equate the relative risk for NGI, RR_NGI_, to RR_HCGI_ calculated in step 1:

$$\begin{array}{ll} {\mathrm{RR}}_{\mathrm{NGI}} & {=\mathrm{RR}}_{\mathrm{HCGI}}\\ & =\left({\mathrm{NS}}_{\mathrm{NGI}}{+\mathrm{SAI}}_{\mathrm{NGI}}\right)/{\mathrm{NS}}_{\mathrm{NGI}}{=\mathrm{RR}}_{\mathrm{HCGI}} \end{array}$$

or,

$${\mathrm{SAI}}_{\mathrm{NGI}}{=\mathrm{NS}}_{\mathrm{NGI}}\times \left({\mathrm{RR}}_{\mathrm{HCGI}}-1\right)$$

After the 1986 tolerable illness risk level has been translated to a new illness risk level that is compatible with the NEEAR study data there is a second step in the process. That step is to convert the new tolerable illness risk level to an Enterococci qPCR CCE (Calibrator Cell Equivalent) guideline value based on the relationship between the GI swimming-associated illness rate and the water quality as measured with a real-time qPCR method.

**Figure 1:**
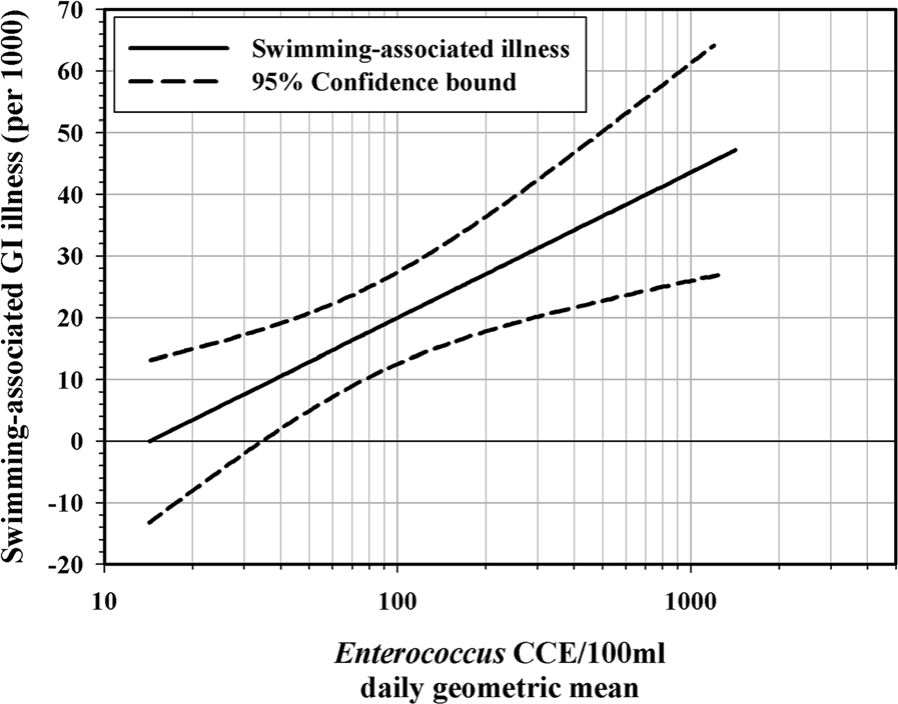
**Swimming-Associated GI illness and Daily Average Enterococcus qPCR CCE.** All Subjects, marine and freshwater beaches combined (Intercept = −27.31, Slope = 23.73)

The relationship between swimming-associated GI illness and water quality measured with the qPCR method has been published previously for freshwater [33] and marine water [34]. The models for these relationships were estimated using an indicator of body immersion-swimming as well as the new measure of water quality, Enterococcus CCE as determined by qPCR, and were adjusted for covariates of concern, such as age, sex, and chronic health conditions, beach and other factors [34,35]. Models were derived from data obtained at beaches that were impacted by point sources of pollution (publicly-owned treatment works, or POTWs) and thus do not necessarily apply to beaches that are impacted only by non-point sources [36]. The slopes and intercepts for the risk levels were generated using a binomial regression model with an identity link function as described previously [35]. Comparison of risk levels with logistic models, and random-effects logistic models resulted in very similar estimates for the fresh and marine data. Therefore, a direct comparison of the risk estimates for fresh and marine beach waters was carried out as described by Altman and Bland [37]. The results indicated that there were significant differences between marine and freshwater in the estimated risk levels only for a limited range of Enterococcus qPCR CCE values (approximately in the range of 100–126 Enterococcus qPCR CCE per 100 mL). Furthermore, a direct test of the slope parameters also shows that there is no difference in the slopes (p = 0.44) or the rate of increase in risk per unit increase in Enterococcus qPCR CCE values, between fresh and marine beaches. A comparison based on the likelihood ratio test (as described in [35]), resulted in the same conclusion. For the likelihood ratio tests, the combined model was estimated with terms that allowed beach specific effects for the indicator term and the swimming term. However, this model was no better than the model with only a single term for each of these parameters (p = 0.19). In effect, there was little evidence for differences in risk estimates obtained from separate models from marine and freshwater beaches and the beach-specific separate models showed no statistical improvement over a single combined model. Therefore, we present risk levels based on the combined model (Figure 1).

The relationship between swimming-associated GI illness and water quality measured with the Enterococcus qPCR method can be described by the equation:

$${\mathrm{SAI}}_{\mathrm{NGI}}=a+b\times {\text{log}}_{10}\;\mathrm{of}\;\mathrm{the}\;\mathrm{indicator}\;\mathrm{density}.$$

Thus, the combined model is:

$${\mathrm{SAI}}_{\mathrm{NGI}}=-27.31+23.73\times {\text{log}}_{10}{\mathrm{ENT}}_{\mathrm{qPCR}.}$$

This model (Figure 1) provides a framework for converting a translated tolerable illness risk level to an Enterococcus qPCR CCE guideline value.

## Results and discussion

The overall non-swimmer illness rates were 14 HCGI illnesses per thousand from the earlier EPA studies and 63 NGI illnesses per thousand from the NEEAR study (note that this includes waders with non-swimmers since the two groups are not statistically different in terms of baseline risk). This indicates a ratio of 4.5 contemporary NGI illnesses to historical, pre-1986 HCGI illnesses among the respective non-swimmer populations.

**Figure 2:**
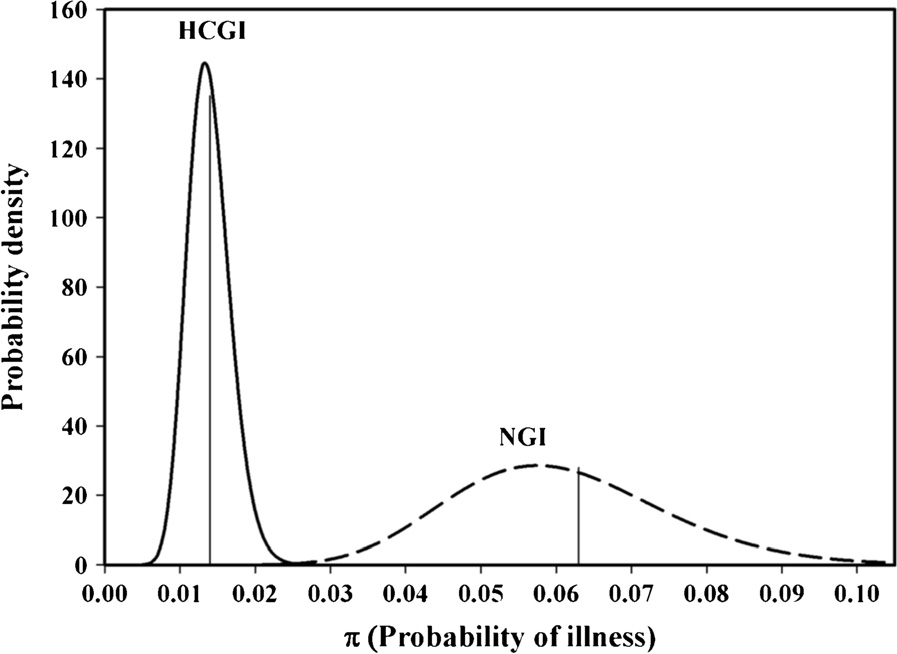
Beta binomial models for the distribution among beaches of the incidence of HCGI and NGI for non-swimmers.

Results of estimating the distribution of background rates of NGI and historical HCGI among beaches (Figure 2) confirm this and allow an evaluation of their relative precisions as well as precision of the ratio between the background incidences of contemporary NGI and pre-1986 HCGI. MCMC estimation indicate mean HCGI incidence to have been 0.014 with a 95% credible interval of 0.012 to 0.018 among non-swimming beach-goers and mean NGI incidence of 0.063 with a 95% credible interval of 0.049 to 0.106. The ratio of NGI to historical HCGI is estimated at 4.4, again based on MCMC simulation, not significantly different from 4.5 (p = 0.94) as calculated above. The 95% credible interval for this risk ratio is 3.2 to 7.7.

One expects baseline rates of illness to vary among the beach-going, but non-swimming population from one location to the next. This variation may be influenced by numerous factors, including illness among the general population at large, non-swimming related exposure at the beach (eating, drinking, beach-goer density, etc.), types of environs from which beach-goers are drawn (urban, rural, suburban, industrial, commercial, etc.). Figure 2 indicates that the incidence of contemporary NGI is more variable than that of HCGI observed pre-1989, but this is largely, if not entirely, due simply to the fact that the definition of NGI is broader and thus has a higher incidence. Based on the distributions shown in Figure 2, 90% of the time actual background incidence rates of HCGI among non-swimmers would have varied beach-by-beach between 10 and 20 cases per thousand, compared to 40 to 90 cases per thousand for NGI, roughly the same two-to-one range, within limits of precision, for either illness. This at least offers a degree of confirmation that the variability of background illnesses beach-to-beach among non-swimmers is typical, either as observed prior to 1986 with respect to the HCGI case definition or more recently with NGI.

The 1986 Criteria for recreational waters implied two different maximum SAI risk levels for freshwater or marine water beaches. For our purposes, we take the lower of the two risks of 8 cases per thousand as the more protective of public health. An equivalent criterion for NGI may then be calculated as:

$$\mathrm{Step}1:{\mathrm{RR}}_{\mathrm{HCGI}}=\left(14+8\right)/14=1.57$$

$$\mathrm{Step}2:{\mathrm{SAI}}_{\mathrm{NGI}}=63\times \left(1.57-1\right)=36$$

Substituting 36 in the equation for SAI_NGI_, above, gives

$${\mathrm{SAI}}_{\mathrm{NGI}}=36=-27.31+23.73\times {\text{log}}_{10}\left({\mathrm{ENT}}_{\mathrm{qPCR}}\right)$$

giving, log _10_(ENT_qPCR_) = 2.668,  *or* ENT_qPCR_ = 466.

Proceeding from the scenario of a common tolerable risk level for both marine and freshwater environments, then, our example of a qPCR criterion will follow from a tolerable risk of 36 swimming-related cases of NGI per 1000 swimmers. This was shown above to be equivalent to 8 HCGI cases per 1000 swimmers, as specified in the 1986 freshwater criteria. Setting the swimming-related risk of NGI in the provisional model to 36 per thousand would imply an Enterococcus *qPCR CCE* geometric mean criterion of 466 cell equivalents per 100 mL for both freshwater and marine beaches.

The translation algorithm presented here is a straightforward approach for linking data reported in 1986 to data collected in the NEEAR study during epidemiological studies conducted between 2002 and 2009. This risk based approach used relative risk levels from the early and more recent EPA studies which had dissimilar case definitions to describe gastrointestinal illness in the two non-swimming populations to link the different illness frequencies to a common relative risk that was described by a simple translation factor. The development of new health data using a new case study definition, a new method for describing water quality and a more contemporary approach to analyzing epidemiological data in the NEEAR study presents a significant challenge to reconciling the new approach to the approach that was used over 25 years ago, which differed in the use of these three elements. The advantage of the current approach is that it can be used with any database that presents a relationship between swimming associated illness and water quality as measured with any valid indicator that might serve as a guideline. Given such a relationship, an acceptable risk level that is already in place or one that has been selected by consensus can be used to translate a prior risk level to one that is appropriate for the relationship in question and to convert that risk level to an indicator guideline value

## Conclusions

A change in case definition in the US EPA epidemiological studies conducted in the 1970–80 and 2002–09 years resulted in an increase in the non-swimming illness rate by a factor of 4.5.

Equivalent risk levels for the illness rates associated with the different case definitions were developed through the application of a consistent relative risk of 1.57.

The new tolerable illness risk level can be used with the regression model that describes the relationship between swimming-associated gastrointestinal illness and water quality measured with a molecular indicator to determine the Enterococcus qPCR density related to an illness rate of 36 per 1000 swimmers or any other tolerable risk level.

## Competing interests

The authors declare that they have no competing interests.

## Authors’ contributions

APD designed and implemented the pre-1986 USEPA studies and was primarily responsible for their interpretation and presentation in this manuscript. TJW was primarily responsible for modeling health data from the NEEAR study and for interpreting and characterizing NEEAR health endpoints for this manuscript. LJW undertook the statistical analysis and took primary responsibility for the manuscript. All authors participated in conceptualizing, drafting and refining the manuscript and approved the final version for publication.

## Pre-publication history

The pre-publication history for this paper can be accessed here:

http://www.biomedcentral.com/1471-2458/13/459/prepub
